# Supplementary material for: Automated 360-degree goniophotography with the NIDEK Gonioscope GS-1 for glaucoma
Source: PLoS One. 2023 Mar 7;18(3):e0270941. doi: 10.1371/journal.pone.0270941 (PMC9990915; doi:10.1371/journal.pone.0270941)
Supplement: S1 File — (DOCX) [file pone.0270941.s001.docx]

CLINICAL STUDY PROTOCOL

# NIDEK Gonioscope GS-1 for Glaucoma Study Number

18-00429

# Investigational Product

NIDEK Gonioscope GS-1

# Clinical Phase

Phase I

# Protocol Version

January 11, 2019

Update: 06 JAN 2020

# Table of Contents

[Synopsis 3](#_TOC_250026)

[Primary Objective 3](#_TOC_250025)

[Study Duration ............................................................,. 3](#_TOC_250024)

[Study Design 3](#_TOC_250023)

[Study Population 3](#_TOC_250022)

[Number of Participants 3](#_TOC_250021)

1. [- Introduction 4](#_TOC_250020)
   1. [Introductory Statement 4](#_TOC_250019)
2. [- Background 4](#_TOC_250018)
3. [- Rationale/Significance 5](#_TOC_250017)
   1. [Problem Statement. 5](#_TOC_250016)
   2. [Potential Risks 5](#_TOC_250015)
4. - Study Objectives 6
   1. Primary Objective 6
   2. Primary Outcome Variables… 6
5. [- Study Design 6](#_TOC_250014)
   1. [Number of Study Sites 6](#_TOC_250013)
   2. [Study Population 6](#_TOC_250012)
   3. [Number of Participants 6](#_TOC_250011)
   4. [Eligibility Criteria/Vulnerable Populations 6](#_TOC_250010)
6. [- Methods 7](#_TOC_250009)
   1. [Study procedure 7](#_TOC_250008)
   2. [Device Specifications 7](#_TOC_250007)
   3. [Informed Consent 8](#_TOC_250006)
   4. [Screening 8](#_TOC_250005)
   5. [Recruitment, Enrollment and Retention 8](#_TOC_250004)
   6. [Removal of subjects 8](#_TOC_250003)
   7. [Primary Analyses 8](#_TOC_250002)
7. [- Trial Administration 9](#_TOC_250001)
   1. [Ethical Considerations 9](#_TOC_250000)
   2. Institutional Review Board (IRB) Review 9
   3. Subject Confidentiality 9
   4. Data Collection 9
   5. Data Storage/Security 9
   6. Study Monitoring 9
   7. Data Safety Monitoring Plan 10
   8. Study Modification… 10
   9. Funding Source 10

# Synopsis

# Primary Objective

To compare the accuracy, reliability, and repeatability of the NIDEK Gonioscope GS-1 (NIDEK, Gamagori, Japan) with conventional gonioscopy and ultrasound biomicroscopy in evaluating the iridocorneal angle in glaucoma and glaucoma suspect patients.

# Study Duration

The study will continuously enroll patients until goal enrollment.

# Study Design

This is a prospective, non-randomized, cross-sectional, experimental study.

# Study Population

We aim to enroll patients diagnosed with glaucoma or who are glaucoma suspects. There will be no enrollment restrictions based on gender, ethnic origin or HIV status and the study population will reflect the demographics of NYU Department of Ophthalmology.

# Number of Participants

60 patients (120 eyes) will be enrolled in this study after an initial ad-hoc power calculation was performed to determine necessity to reach statistical significance.

# - Introduction

# Introductory Statement

The NIDEK Gonioscope GS-1 captures iridocorneal angle images using an optical gonioprism and a built-in image sensor without the use of mydriatic agents. Through the use of a built-in rotator unit, a color circumferential image of the iridocorneal angle and its peripheral area is captured. The captured image can be stored in the device’s internal memory, printed onto paper, or transmitted to external equipment.

# - Background

Gonioscopy is a vital part of the ophthalmologic examination, as it allows clinicians to evaluate the iridocorneal angle of the eye. The iridocorneal angle contains the orifice to the draining pathway of the aqueous humor from the anterior chamber into the canal of Schlemm. More acute, or “narrow,” angles can put patients at risk for glaucoma due to obstruction of the route of the fluid into the trabecular meshwork. Accumulation of pigment or inflammatory cells can also lead to obstructions of the drainage system and consequent increase in intraocular pressure. Gonioscopy allows clinicians to assess the width of the angle as well as look for any structural deformation or accumulation of material.

The goniolens is a specially designed lens to allow visualization of the angle that is placed on the patient’s cornea by the clinician during the ocular examination. When used in conjunction with a slit lamp, it gives the clinician a view of the iridocorneal angle, which is otherwise masked by the ocular configuration. Although it is considered the gold standard for assessment of the iridocorneal angle, gonioscopy can be difficult to perform. [1] The evaluation of the width of the iridocorneal angle is subjective and requires clinical experience. [1] As such, there is some inter-operator variability in the results obtained using this method.

Due to the time and skill required to obtain an accurate gonioscopic image on a conventional slit lamp, there has been interest in developing alternative methods to obtain quick, accurate, and reliable images of the iridocorneal angle. These alternative methods have involved the use of both new and existing technologies. For instance, currently available 3D Corneal and Anterior Segment Optical Coherence Tomography (3D CAS-OCT) has been used for this purpose. A study investigating the utility of 3D CAS-OCT in evaluating anterior segment parameters found a high degree of intra-grader repeatability, inter-grader repeatability, and correlation with conventional gonioscopy. [2]

With the goal of someday replacing conventional gonioscopy, a number of imaging devices have been developed specifically to assess anterior segment parameters. The Pentacam-Scheimpflug camera uses the Scheimpflug optical principle to photograph parts of the anterior segment that are not directly in line with the camera’s aperture. [3] The camera takes up to 50 slit images of the anterior segment in 2 seconds and uses software to construct a 3D image. [3] The benefit of this technology is that it requires little operator experience and begins scanning automatically once proper alignment has been achieved. [3] However, Pentacam-Scheimpflug only quantitatively estimates the width of the angle and does not directly visualize it. [3] It therefore cannot be used as a replacement for conventional gonioscopy.

Orbscan Scanning-Slit Topography is a technology that scans the entire surface of the cornea, the iris, and the lens using a slit-scanning beam. [3] By mapping both the posterior

surface of the cornea and the iris, Orbscan calculates an estimation of the iridocorneal angle. [3] However, like Pentacam-Scheimpflug, it does not directly visualize the angle. Paradigm P60 ultrasound biomicroscope can obtain images of the iridocorneal angle and has been used for decades in the form of A- and B-scans. [3] However, in order to obtain an image, it requires direct contact with the eye, which is uncomfortable for the patient, and is highly operator- dependent. [3]

The clinical utility of an imaging device that can produce quick, accurate, and reliable images of the iridocorneal angle is clear. If the NIDEK Gonioscope GS-1 can produce images that are of comparable quality to digital images obtained from a goniolens and slit lamp, it has the potential to increase the speed of clinical examination in glaucoma clinics and improve workflow by replacing conventional gonioscopy.

# - Rationale/Significance

# Problem Statement

Although gonioscopy plays an important role in glaucoma diagnosis and treatment, repeatability and reliability are user-dependent when a conventional goniolens is used. Furthermore, during examination, the goniolens must be manipulated to a significant degree to obtain full visualization of the iridocorneal angle. This study evaluates the reliability and accuracy of NIDEK Gonioscope GS-1 in obtaining images of the iridocorneal angle. The NIDEK Gonioscope GS-1 captures 360° images of the anterior segment, including the iridocorneal

angle, using a light source, multi-mirror prism, and a sensor. This device does not require use of a slit lamp nor manipulation of a goniolens.

# Potential Risks

Because the multi-mirror prism touches the surface of the eye it carries a risk of infection. We will perform high level disinfection or low temperature sterilization (EOG sterilization) after any use of the prism to reduce this risk. Additionally, all exposed surfaces near the eye, as well as the chin rest and forehead rest of the instrument, will be cleaned with alcohol before participants are examined. The multi-mirror prism is made from cycloolefin polymer (COP) resin. *In vitro* cytotoxicity assays found that concentrations of up to 100% COP did not obstruct colony formation of V79 Chinese hamster lung cells. Ocular irritation tests showed that COP does not irritate the cornea, iris, or conjunctiva of rabbits.

The prism may cause a foreign body sensation to the patient during the capture of iridocorneal angle image which may cause some discomfort.

Ultrasound biomicroscopy is an FDA-approved technology that uses an ultrasound probe to visualize intraocular structures, including the iridocorneal angle. The ultrasound probe does not directly contact the eye, but rather a balloon filled with water is put in contact with the eye. The probe contacts the water balloon. There is risk of some minor patient discomfort during ultrasound biomicroscopy.

# - Study Objectives Primary Objective

To compare the accuracy, reliability, and repeatability of the NIDEK Gonioscope GS-1 (NIDEK, Gamagori, Japan) with ultrasound biomicroscopy and conventional gonioscopy in evaluating the iridocorneal angle in glaucoma or glaucoma suspect patients. Repeatability refers to the ability of 2 separate ophthalmologists to identify the same iridocorneal angle width from each NIDEK image.

# Primary Outcome Variables

Images obtained by the NIDEK Gonioscope GS-1 will be compared with ultrasound biomicroscopy images and digital gonioscopic images obtained using a goniolens and slit lamp to determine if the NIDEK Gonioscope GS-1 can accurately and reliably capture relevant findings. To determine the accuracy of the NIDEK Gonioscope GS-1, we will use ANOVA to determine if the iridocorneal angle measurements obtained from the NIDEK Gonioscope GS-1 are statistically similar to measurements obtained from ultrasound biomicroscopy and standard gonioscopy. To determine inter-observer reproducibility when using this device, two observers will examine each image obtained from NIDEK Gonioscope GS-1 and separately determine the iridocorneal angle. Inter-observer reproducibility will be determined using Kappa's statistics and intraclass correlation coefficient. Lastly, we will determine if the time it takes to obtain images from the NIDEK Gonioscope GS-1 is statistically different from the time it takes to obtain images by conventional gonioscopy. This will be done via ANOVA.

# - Study Design

# Number of Study Sites

This study will take place at the New York University Langone Health Department of Ophthalmology and Bellevue Hospital.

# Study Population

This study will enroll patients that have been diagnosed with glaucoma or who are glaucoma suspect.

# Number of Participants

Following a preliminary data collection, we were able to estimate the sample size needed to reach statistically significant results. Assuming that the manual gonioscopy and automated gonioscopy GS-1 have an equal probability of recording a diagnosis, Cohen's Kappa of 0.34 was measured. To detect a fair agreement between the two modalities, while achieving 80% power and 5% type I error, we will need to collect data from 94 eyes. To account for the portion of images that are uninterpretable due to poor quality (10-20%), we asses a total of 120 eyes (60 participants) would be satisfactory for us to estimate the GS-1 as a diagnostic tool accurately.

# Eligibility Criteria/Vulnerable Populations

Inclusion criteria:

-Aged 18 and older

-Diagnosed with glaucoma or glaucoma suspect Glaucoma Suspect

- - 1. Glaucomatous optic neuropathy as described in the glaucoma patients below
    2. AND/OR ocular hypertension (IOP > 21 mm Hg)
    3. Normal visual field Glaucoma Patients

1. Clinical characteristics of glaucoma: ONH abnormalities: global rim thinning, rim notch, or disc hemorrhage; RNFL defect

Typical glaucomatous field loss in reliable VF, reproducible glaucoma hemifield tests labeled outside normal limits on at least two consecutive tests.

-Both eyes will be included, except in cases where only one eye meets study criteria

Exclusion criteria:

-Corneal opacities (scars, edema, etc.)

-Pregnant or planning to become pregnant

-Inability to fixate gaze

# - Methods

# Study Procedure

Subjects who meet eligibility criteria will be identified by study team and will be consented for the study. During their usual ophthalmology visit, all subjects will undergo conventional gonioscopy, ultrasound biomicroscopy, and gonioscopy with NIDEK Gonioscope GS-1. The NIDEK Gonioscope GS-1 will only be used on patients who are in the study. The experimental part of the visit using the NIDEK Gonioscope GS-1 should take approximately 10 minutes,

For conventional gonioscopy, the clinician may obtain digital images of the iridocorneal angle using a slit lamp and goniolens. For ultrasound biomicroscopy, the clinician may obtain ultrasound images that will then be stored within the biomicroscope device. For gonioscopy with NIDEK Gonioscope GS-1, the device may obtain and store images of the iridocorneal angle.

Following collection of the images, one ophthalmologist may examine images obtained from conventional gonioscopy and determine the width of the iridocorneal angle. The same ophthalmologist will determine the width of the iridocorneal angle from the ultrasound biomicroscopy images. This ophthalmologist will be masked to patient names and diagnoses. A second and potentially third ophthalmologists may examine images obtained from NIDEK Gonioscope GS-1 and determine the width of the iridocorneal angle. These ophthalmologists will also be masked to patient names and diagnoses.

Statistical analysis will be performed to determine the association between the findings of iridocorneal angle width in conventional gonioscopy images, ultrasound biomicroscopy images, and NIDEK Gonioscope GS-1 images of each patient. Further statistical analysis will be performed to determine the inter-observer repeatability of the findings of the two ophthalmologists who examined the images from NIDEK Gonioscope GS-1.

# Device Specifications

The NIDEK Gonioscope GS-1 obtains gonioscopic images without using a goniolens or slit lamp. This device captures 360° images of the anterior segment, including the iridocorneal angle, in a single examination. The 16-face multi-mirror prism projects light onto the patient's iridocorneal angle and its peripheral area, and uses an image sensor to generate the image. A coupled gel will be applied to the prism tip prior to using the device. This gel reduces the refraction angle between the patient's cornea and the prism and moderates the variation in each patient's corneal shape.

Although the NIDEK Gonioscope GS-1 is not currently an FDA-approved device, it is a non- significant risk device under the criteria of 21 CFR 812.3 (m):

- - 1. It is NOT intented as an implant and does NOT present a potential for serious risk to the health, safety, or welfare of a subject
    2. It is NOT purported or represented to be for a use in supporting or sustaining human life and does NOT present a potential for serious risk to the health, safety, or welfare of a subject
    3. It is for a use of substantial importance in diagnosing, curing, mitigating, or treating disease, or otherwise preventing impairment of human health and does NOT present a potential for serious risk to the health, safety, or welfare of a subject

# Informed Consent

The study will take place during the course of a single visit. Subjects will be approached by a study team member during a routine clinical visit and will be asked to participate. They will be given ample time to read, understand, and provide informed consent.

Documentation of the informed consent process will be used to ensure that the appropriate questions have been asked to the prospective participant.

# Screening

Eligible subjects will be identified from the clinic through chart review and approached for consent.

# Recruitment, Enrollment and Retention

Subjects will be identified from NYU Department of Ophthalmology and Bellevue Hospital Ophthalmology clinic. Eligibility will be determined based on the results of a patient's eye examination, and qualified subjects will be offered to participate in the study.

# Removal of subjects

Subjects may withdraw their consent for participation in this research study at any time. Any identifiable research or medical information recorded for, or resulting from, subject participation in this research study prior to the date that subject formally withdrew consent may continue to be used and disclosed by the investigators for the purposes described above.

# Primary Analyses

The analysis of the width of the iridocorneal angle will be done according to both the Scheie and Shaffer grading systems. Relationships between results obtained from conventional gonioscopy, ultrasound biomicroscopy, and NIDEK Gonioscope GS-1 will be evaluated by linear and quadratic models, as well as more advanced statistical methods.

# - Trial Administration

# Ethical Considerations

This study will be conducted in compliance with the protocol approved by the IRB, GCP guidelines, and applicable NYU Langone Health and federal regulatory requirements. No deviation from the protocol will be implemented without prior review and approval of the IRB,

except where it may be necessary to eliminate an immediate risk to a participant. In such case, the deviation will be reported to the IRB according to its policies and procedures.**7.2 Institutional Review Board (IRB) Review**

This protocol, associated consent form, and any amendments will be submitted to an IRB in agreement with local legal prescriptions, for formal approval of study conduct. The decision of the IRB concerning the conduct of the study will be made in writing to the investigator.

# Subject Costs and Payments

There will be no costs for subjects or payments to subjects when participating in this study.

# Subject Confidentiality

Information about study participants will be kept confidential and managed according to the requirements of the Health Insurance Portability and Accountability Act of 1996 (HIPAA). In accordance with these regulations, participants will sign authorization of the following:

- The protected health information (PHI) that will be collected
- Who will have access to that information and why
- Who will use or disclose that information
- The rights of a research participant to revoke their authorization for use of their PHI

In the event that a participant revokes authorization to collect or use PHI, the investigator retains the ability to use all information collected prior to the revocation of authorization.

Data will be collected after the participant has signed an informed consent form and completed their visit. It will be collected from the medical record, including Epic, and other sources. The collected data include demographics, clinical information and testing results. Data will be collected only by members of the research team and stored in accordance with Federal and University regulations. Research related materials will be retained and stored following NYU policy (a minimum of seven years following study completion). We will use RedCap. ICF's will also be retained for seven years. Paper-based records will be kept in a secure location within a double

locked room and only be accessible to personnel involved in the study. Computer-based files will only be made available to personnel involved in the study through the use of access privileges and passwords, granted prior to access to any study-related information residing within NYU firewall. Data from sub-sites will be periodically transferred to the central site through a secured network connection or by encrypted and password protected hard drives

# Data Collection

Data will be collected only after the participant has signed an informed consent form. The collected data will include demographics, clinical information and goniometry results. Data management will include steps that will ensure confidentiality and security of the data. Data will be collected only by members of the research team and stored in accordance with Federal and University regulations. The data will be de-identified prior and the key will be accessible only by study personnel. Research related materials will be retained and stored following NYU Langone Health policy.

# Data Storage/Security

Research related materials will be retained and stored in a secure MCIT drive following NYU Langone Health policy. Identifiable health information will not be reused or disclosed to any other person or entity, except as required by law or for authorized oversight of the research study.

# Study Monitoring

The Principal Investigator will oversee the safety of the patients. The safety monitoring will include careful assessment and appropriate reporting of adverse events as noted above.

# Data Safety Monitoring Plan

**T y p e s o f D a t a o r E v e n t s**

The following aspects will be specifically monitored: enrollment and retention, data collection, confidentiality, breaches from the study protocol or any adverse event. Adverse events include injury to subjects from the NIDEK Gonioscope GS-1, as described in Section 3.2 of this document.

# R e s p o n s i b i l i t i e s a n d R o l e s f o r G a t h e r i n g , E v a l u a t i n g a n d M o n i t o r i n g t h e D a t a

- - - All study team members will be responsible for reporting data related to unanticipated problems and adverse events
    - If injury occurs to a participant, the ophthalmologist will immediately notify the PI and Gadi Wollstein, MD. A report will then immediately be sent to the IRB from one of these 2 people.
    - Gadi Wollstein, MD will verify data accuracy, by reading it and discussing with co- investigators, once per quarter
    - Gadi Wollstein, MD will verify compliance with the protocol, by comparing actual practice to the protocol, once per quarter.

# I n f o r m a t i o n A b o u t t h e M o n i t o r i n g E n t i t y

- DMC consisting of 3 members:
- Joel Schuman, MD (PI), Gadi Wollstein, MD and Zena Moore (Research Coordinator). Contact information can be found elsewhere in the IRB application

# R e p o r t i n g A d v e r s e E v e n t s a n d U n a n t i c i p a t e d P r o b l e m s t o t h e M o n i t o r i n g E n t i t y

- All adverse events will be reported immediately to IRB
- Reporting will be done via email
- Gadi Wollstein, MD or study team members will prepare and submit the form.

# A s s e s s m e n t s

- Monitoring entity will review and assess the data or events captured under the Data Monitoring Plan quarterly. Monitoring entity will review data and events according to assessment criteria for “unanticipated problems involving risks to participants or others” (i.e., as to whether they are unexpected, related and harmful)

# C r i t e r i a f o r A c t i o n

- Any breach of data confidentiality or injury to participants will trigger action
- Study will be stopped if there is injury to a participant

# P r o c e d u r e s f o r C o m m u n i c a t i n g – d i s s e m i n a t i o n o f s a f e t y i n f o r m a t i o n A s A p p r o p r i a t e

- Outcomes of monitoring entity reviews will only be communicated to IRB if an adverse event is found.

The study will be routinely monitored by the PI and the study team during DSMB meetings. The following aspects will be specifically monitored: enrollment and retention, data collection, confidentiality, breaches from the study protocol or any adverse event. The Investigator will also ensure that monitors or other compliance or quality assurance reviewers are given access to all the above noted study-related documents and study related facilities (e.g. diagnostic tests), and have adequate space to conduct the monitoring visit.

Data safety monitoring reviews will occur once per quarter. Adverse events may include loss of data, misplacement of data, or unauthorized access of data. Any and all adverse events will be reported immediately to the IRB by the primary investigator or study personnel by email.

Outcomes of the data safety monitoring reviews will be submitted no less than annually with the continuing review application.

# Study Modification

All study modifications will be submitted and approved by the IRB prior to implementing changes.

# Funding Source

This study will be funded by the NYU Department of Ophthalmology.

References

1. Campbell, Peter, et al. "Repeatability and comparison of clinical techniques for anterior chamber angle assessment." *Ophthalmic and Physiological Optics* 35.2 (2015): 170-178.
2. Wang, Dandan, et al. "A new 3-Dimensional (3D) method for assessment of the anterior chamber angle." *Investigative Ophthalmology & Visual Science* 55.13 (2014): 4836- 4836.
3. Konstantopoulos, Aristides, Parwez Hossain, and David F. Anderson. "Recent advances in ophthalmic anterior segment imaging: a new era for ophthalmic diagnosis?." *British Journal of Ophthalmology* 91.4 (2007): 551-557.
