# Supplementary material for: Automated 360-degree goniophotography with the NIDEK Gonioscope GS-1 for glaucoma
Source: PLoS One. 2023 Mar 7;18(3):e0270941. doi: 10.1371/journal.pone.0270941 (PMC9990915; doi:10.1371/journal.pone.0270941)
Supplement: S4 File — (PDF) [file pone.0270941.s004.pdf]

subject	eye	quadrant	shaffer_doc1	shaffer_doc2	shaffer_doc3
1	OD	S	4	4	4
1	OD	N	4	4	4
1	OD	I	4	4	4
1	OD	T	4	4	4
1	OS	S	4	4	4
1	OS	N	4	4	4
1	OS	I	4	4	4
1	OS	T	4	4	4
2	OD	S	4	4	0
2	OD	N	4	4	2
2	OD	I	4	4	2
2	OD	T	4	4	3
2	OS	S	4	4	0
2	OS	N	4	4	2
2	OS	I	4	4	2
2	OS	T	4	4	3
4	OD	S	4	4	2
4	OD	N	4	4	2
4	OD	I	4	3	2
4	OD	T		4	2
4	OS	S	4	4	2
4	OS	N	4	4	2
4	OS	I	4	4	2
4	OS	T	4	4	2
5	OD	S	4	4	3
5	OD	N	4	4	3
5	OD	I	4	4	3
5	OD	T	4	4	3
6	OD	S	4	4	4
6	OD	N	4	4	4
6	OD	I	4	4	4

**6**	OD	T	4	4	4
**6**	OS	S	4	4	4
**6**	OS	N	4	4	4
**6**	OS	I	4	4	4
**6**	OS	T	4	4	4
**7**	OD	S	4	4	4
**7**	OD	N	4	4	4
**7**	OD	I		4	4
**7**	OD	T	4	4	4
**7**	OS	S	4	4	4
**7**	OS	N	4	4	4
**7**	OS	I	4	4	4
**7**	OS	T	4	4	4
**8**	OD	S		4	
**8**	OD	N	4	4	
**8**	OD	I	4	4	
**8**	OD	T	4	4	
**8**	OS	S	4	4	
**8**	OS	N	4	4	
**8**	OS	I		0	
**8**	OS	T	4	4	
**9**	OD	S	4	4	4
**9**	OD	N	4	4	4
**9**	OD	I	4	4	4
**9**	OD	T	4	4	4
**10**	OD	S	4	4	3
**10**	OD	N	4	4	3
**10**	OD	I	4	4	3
**10**	OD	T	4	4	3
**10**	OS	S	4	4	2
**10**	OS	N	4	4	2
**10**	OS	I	4	4	2

**10**	OS	T	4	4	2
**11**	OD	S	4	4	3
**11**	OD	N		4	3
**11**	OD	I		4	3
**11**	OD	T	4	4	3
**12**	OD	S	4	4	4
**12**	OD	N	4	4	4
**12**	OD	I	4	4	4
**12**	OD	T	4	4	4
**12**	OS	S	4	4	4
**12**	OS	N	4	4	4
**12**	OS	I	4	4	4
**12**	OS	T	4	4	4
**13**	OD	S		4	
**13**	OD	N		4	
**13**	OD	I		4	
**13**	OD	T		4	
**13**	OS	S		4	
**13**	OS	N		4	
**13**	OS	I	4	4	
**13**	OS	T		4	
**15**	OD	S			
**15**	OD	N			
**15**	OD	I			
**15**	OD	T			
**15**	OS	S	4	4	4
**15**	OS	N		4	4
**15**	OS	I	0	4	3
**15**	OS	T	0	4	3
**16**	OD	S		0	3
**16**	OD	N		3	3
**16**	OD	I		2	

**16**	OD	T			
**16**	OS	S		3	4
**16**	OS	N		4	3
**16**	OS	I		4	3
**16**	OS	T		3	4
**17**	OD	S		4	2
**17**	OD	N	4	4	2
**17**	OD	I	4	4	2
**17**	OD	T		4	2
**17**	OS	S		4	2
**17**	OS	N	4	4	2
**17**	OS	I	4	4	2
**17**	OS	T		4	2
**18**	OD	S	4	4	4
**18**	OD	N	4	4	4
**18**	OD	I	4	4	4
**18**	OD	T		4	4
**18**	OS	S		4	4
**18**	OS	N	4	4	4
**18**	OS	I	4	4	4
**18**	OS	T	4	4	4
**19**	OD	S	4	4	3
**19**	OD	N	4	4	3
**19**	OD	I		2	3
**19**	OD	T		2	
**19**	OS	S	4	4	3
**19**	OS	N	4	4	3
**19**	OS	I	4	4	3
**19**	OS	T		4	3
**20**	OD	S			
**20**	OD	N			
**20**	OD	I			

**20**	OD	T			
**20**	OS	S			
**20**	OS	N			
**20**	OS	I			
**20**	OS	T			
**21**	OD	S		1	
**21**	OD	N			
**21**	OD	I		3	
**21**	OD	T		2	
**21**	OS	S		3	3
**21**	OS	N		3	3
**21**	OS	I	0	3	3
**21**	OS	T	0	3	3
**22**	OD	S	4	4	4
**22**	OD	N	4	4	4
**22**	OD	I	4	4	4
**22**	OD	T	4	4	4
**22**	OS	S	4	4	4
**22**	OS	N	4	4	4
**22**	OS	I	4	4	4
**22**	OS	T		4	4
